# Vestibular animal models: contributions to understanding physiology and disease

**DOI:** 10.1007/s00415-015-7909-y

**Published:** 2016-04-15

**Authors:** Hans Straka, Andreas Zwergal, Kathleen E. Cullen

**Affiliations:** Department Biology II, Ludwig-Maximilians-University Munich, Grosshaderner Str. 2, 82152 Planegg, Germany; German Center for Vertigo and Balance Disorders, DSGZ, Ludwig-Maximilians-University of Munich, Munich, Germany; Department of Neurology, Ludwig-Maximilians-University of Munich, Munich, Germany; Department of Physiology, McGill University, Montreal, QC H3A 0G4 Canada

**Keywords:** Otolith organ, Semicircular canal, Sensory–motor processing, Motion perception, Gaze stabilization, Vestibulo-ocular reflex

## Abstract

Our knowledge of the vestibular sensory system, its functional significance for gaze and posture stabilization, and its capability to ensure accurate spatial orientation perception and spatial navigation has greatly benefitted from experimental approaches using a variety of vertebrate species. This review summarizes the attempts to establish the roles of semicircular canal and otolith endorgans in these functions followed by an overview of the most relevant fields of vestibular research including major findings that have advanced our understanding of how this system exerts its influence on reflexive and cognitive challenges encountered during daily life. In particular, we highlight the contributions of different animal models and the advantage of using a comparative research approach. Cross-species comparisons have established that the morpho-physiological properties underlying vestibular signal processing are evolutionarily inherent, thereby disclosing general principles. Based on the documented success of this approach, we suggest that future research employing a balanced spectrum of standard animal models such as fish/frog, mouse and primate will optimize our progress in understanding vestibular processing in health and disease. Moreover, we propose that this should be further supplemented by research employing more “exotic” species that offer unique experimental access and/or have specific vestibular adaptations due to unusual locomotor capabilities or lifestyles. Taken together this strategy will expedite our understanding of the basic principles underlying vestibular computations to reveal relevant translational aspects. Accordingly, studies employing animal models are indispensible and even mandatory for the development of new treatments, medication and technical aids (implants) for patients with vestibular pathologies.

## Introduction

Scientific research on the vestibular system has benefitted from studies on a wide variety of vertebrate species [1]. Systematic investigations of inner ear endorgans in fishes, amphibians and birds, performed over a century ago, revealed many details of the structure/function of this sensory system and established that it is remarkably preserved across vertebrate phylogeny [2, 3]. This organizational conservation emphasizes that the ability to detect and encode body motion for gaze and posture stabilization as well as for orientation and navigation in space is essential across vertebrates [4, 5]. In addition, the almost identical structure of sensory endorgans, neuronal pathways and central circuits in different vertebrates provides the ability to make general conclusions regarding the signaling properties and computational capabilities of the neuronal components of vestibular pathways. Moreover, species-specific features, related to different locomotor strategies/dynamics, particular lifestyles or eco-physiological habitats, offer the opportunity to evaluate the capacity of the system to adapt to new challenges. Thus, understanding the particularities and similarities of vestibular signal processing in different vertebrates have provided an essential opportunity to identify conceptual principles that coincide with the behavioral repertoire and performance. This knowledge has in turn facilitated our understanding of the mechanistic operations required for stabilizing gaze and posture and yielded insight into substrates and processes underlying different pathologies and potential treatments.

Notably, based on their evolutionary proximity to humans, non-human primates have become a standard model for furthering our knowledge of basic vestibular processing and advancing translational research. In addition, advances in the generation of mouse lines with defined genetic backgrounds and/or transgenic lines, such as CRE, combined with viral-based optogenetics have provided new opportunities to probe the functional circuitry of vestibular pathways and gain insight into vestibular diseases or age-related impairments [6–8]. However, it is also important to emphasize that work across a wider variety of different vertebrate models has improved our understanding of vestibular processing. Research encompassing a combination of standard models and less widely used “exotic animals” with particular motion repertoires and/or unique experimental advantages have proven advantageous. This review highlights recent progress that has been made toward understanding the fundamental physiological principles of vestibular processing using different animal models as well as how cellular and circuit properties are altered under pathophysiological conditions.

## Animal models and major topics in vestibular research

### Historical overview

The vestibular endorgans are located within the petrosal bone in close vicinity to the cochlea. This hidden location proximal to the auditory organ and the difficult access considerably delayed our understanding of the functional role of these inner ear organs. It was only in 1824, that Flourens [9] conducted the first systematic behavioral studies on the vestibular system. He discovered that interrupting specific semicircular canals in different vertebrate species, including pigeons and rabbits, produced direction-specific impairments of the equilibrium, walking and head movements. Interestingly, he speculated that the deficits were due to changes in hearing sensitivity, since the semicircular canals were then generally considered as part of the auditory organ. In fact, the vestibular system was only postulated to be a distinct sense independent of hearing in the late 19th century following systematic lesion experiments on frog, pigeon [2] and dog [10]. Both, Goltz [2] and Bechterew [10] concluded that the semicircular canals were distinct organs responsible for posture and equilibrium in three spatial orientations. This view was subsequently confirmed by studies in numerous other species including salamanders, pigeons, cockatoos and rabbits [3]. Concurrently, more theoretically based investigations determined that the semicircular canals sense head rotations [11–13].

As for the semicircular canals, the initially assumed auditory role of otolith organs also remained unchallenged for a long time. It is noteworthy that this historical progression in our understanding is reflected in existing terminology, since otolith literally means “hearing stone”. Theoretically based investigations had deduced that the otolith endorgans sense linear accelerations including head tilts [12, 13], yet experimental work in fish found that removal of the large, solid crystals (otoconia) covering the otolith sensory epithelia [14] significantly impaired their underwater hearing [15]. Experiments in terrestrial vertebrates were required to definitively establish that the otolith organs are responsible for ensuring stable posture and equilibrium in land-based animals [3, 16]. This conclusion was then furthered by experiments in fish indicating that, in this specific group of vertebrates, the saccule/lagena may also serve as a hearing organ [17], thus exerting a dual functional role.

In summary, a comparative approach including studies in vertebrate species from fish to mammals was essential to the progress that was made in the early vestibular research of the 19th century. In particular, the knowledge obtained following experimental lesions of semicircular canals, otoliths, and/or their nerves using different animal models has proven crucial for providing the important insight into species-specific adaptations of the endorgans and variations in their function relative to lifestyle, eco-physiological niche or locomotor dynamics.

### Principles of mechanotransduction and hair cell dynamics

Head motion relative to space is detected and decomposed into individual vector components by semicircular canal and otolith organs [18]. The semicircular canals and otolith organs sense rotation, and linear motion or changes in head position relative to the Earth´s gravitation vector, respectively. The fluid-filled ducts of the semicircular canals enable the detection of angular acceleration by means of the fluid’s inertia relative to sensory epithelia. The mechanistic principle of the otolith organs is based on the inertia of an otolithic structure covering the sensory epithelia [19]. While the spatial arrangement of the bilateral semicircular canals is largely conserved across vertebrates [20], comparative studies in fish, frogs and birds demonstrate that otolith organs can serve an auditory as well as vestibular function as a result of differences in the morpho-physiological properties of hair cells at a particular region of the otolithic epithelium [21]. Thus, in species such as frogs, fish and likely also in mammals, the otolith organs detect changes of the body position relative to the gravity vector as well as substrate/water vibrations [22]. Furthermore, the lagena, an otolith organ that is present in all non-mammalian vertebrates and monotremes [23], likely contributes to the sense of magnetoreception that allows birds to make use of the geomagnetic field for orientation and navigation [24].

Current evidence suggests that vestibular endorgans across species have adapted to changes in the environment and body mass [25], indicating that vestibular processing is influenced by the statistics of natural stimuli encountered in the sensory environment [26]. For example, the transition from water to land-based life has resulted in major changes in the natural sensory environment since the resistive hydrodynamic forces of an aquatic environment effectively dampen self-motion [27]. In addition, the longer and more flexible necks of amniotes can cause faster head movements [28]. Together, these factors suggest that terrestrial amniotes generally experience stimulation at higher amplitudes compared to mostly neckless fish or amphibian species.

During ontogeny, vestibular reflexes are generally present after hatching/birth in precocial animals such as larval fish, amphibian tadpoles or certain avian species or as soon as the respective neural circuitry and/or cellular properties are mature in altricial species [29]. While the developmental onset of otolith function is independent of animal size, semicircular canal functionality critically depends on tube dimensions as predicted by theoretical considerations [30] and verified by experimental data [31–34]. In particular, studies on the small larvae of fish and amphibians established the time course of onset, progression and maturation of motion-evoked semicircular canal-dependent reflexes after embryogenesis [34, 35]. This illustrates a general size dependency of semicircular canal reflexes and an important role for the spatial tuning of otolith-derived extraocular motor responses [36].

Hair cells in the vestibular system have long been a subject of functional studies using various vertebrate species including bullfrog [37, 38], turtle, chinchilla and monkey (reviewed in [39]). While two types of hair cells (type I and II) are present in the inner ear of amniote vertebrates, anamniotes such as fish and amphibians possess only type II hair cells, which are exclusively contacted by bouton-like afferent terminals [39]. The evolutionary appearance of hair cells with a calyx-like afferent synapse (type I hair cells) in amniotes corresponds to a terrestrial lifestyle as well as with the appearance of flexible necks, since as noted above both factors likely contribute to higher frequency and acceleration head movements. This view concurs with the fact that responses of type I hair cells are considerably more dynamic than those of type II hair cells [39]. However, despite the absence of type I hair cells, frog otolith hair cells exhibit a similarly broad spectrum of response dynamics, suggesting that a calyx-like structure is no prerequisite for encoding high dynamic motion stimuli [40]. Recent studies in chinchilla and rodents [41] suggest that more detailed investigations of the complex type I hair cell calyx are required to fully understand its physiological implications/advantages.

### Neural encoding strategies in the peripheral vestibular system

**Fig. 1:**
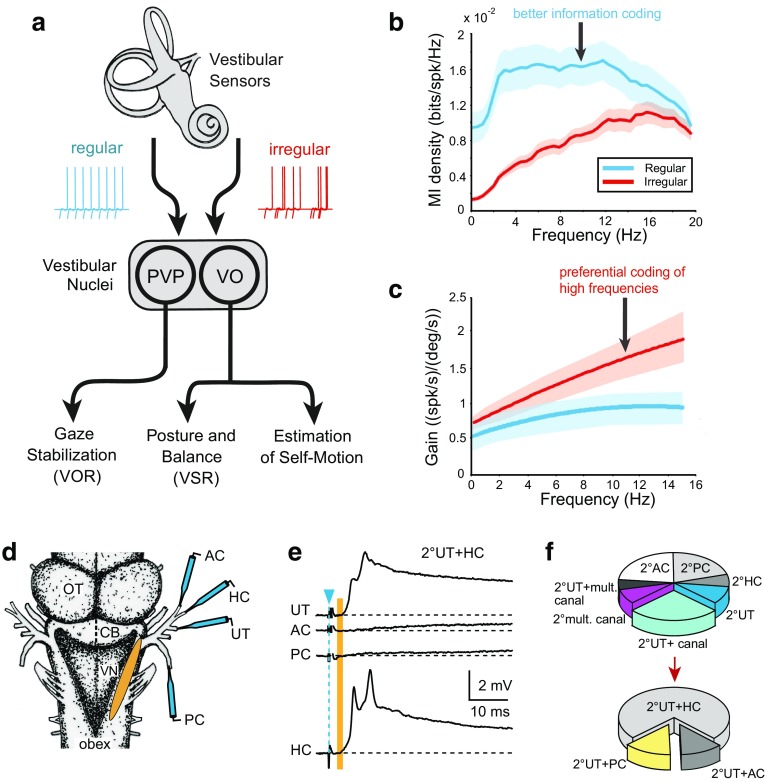
Vestibular sensory–motor signal processing. **a** Schematic illustrating the two channels of input from labyrinthine nerve afferents (regular, irregular) and main subclasses of central vestibular neurons (PVP, VO) underlying vestibular reflexes (VOR, VSR) and self-motion perception. **b**, **c** Gain and mutual information density for regular and irregular vestibular afferent fibers. Population-averaged mutual information density curves (±SEM, **b**) and gains (±SEM, **c**) during random head rotations as function of frequency. **d**–**f**, Convergence of monosynaptic semicircular canal and otolith signals in frog 2°VNs; **d** schematic of an isolated frog whole brain depicting the electrical stimulation of individual labyrinthine nerve branches and central vestibular recording area (orange); **e** 2°VNs, identified by monosynaptic (vertical orange bar) EPSPs (UT + HC) following separate stimulation (blue arrowhead) of the AC, HC, PC and UT nerve branches; **f** convergence pattern of utricular and semicircular canal nerve afferent inputs in identified 2°VNs. *AC, PC, HC* anterior, posterior vertical, horizontal semicircular canal, *AP, BP* amphibian, basal papilla, *CB* cerebellum, *LA* lagena, *OT* optic tectum, *PVP* position-vestibular-pause neuron, *UT* utricle, *VN* vestibular nuclei, *VO* vestibular-only neuron, *VOR* vestibulo-ocular reflex, *VSR* vestibulo-spinal reflex. **b**, **c**, **e**, **f** are based on data from [46] and [70], respectively

Previous studies have shown that afferents supplied by type I hair cells, which are found in amniotes, tend to be more irregular (Fig. 1a) in their resting discharges than afferents that contact type II hair cells [39]. This obvious dichotomy is compatible with the differential composition of ion conductances in vestibular ganglion cells (reviewed in [27]). Irregular afferents with calyx-like terminals have higher sensitivities and are better suited for the processing of natural motion stimuli in comparison to regular afferents (Fig. 1b) [26]. However, while there are clear physiological differences between irregular and regular afferent fibers in amniotes, it is notable that afferent response dynamics also vary in anamniotes. For example, a subclass of semicircular canal afferents in toadfish encodes angular acceleration [42], even though anamniotes have only type II hair cells.

Over the range of frequencies typically experienced during everyday behaviors (i.e., up to 20 Hz), semicircular canal afferents encode head velocity while otolith afferents encode linear acceleration in mammals ranging from mice to primates [43–45]. Quantification of afferent responses in primates has revealed important differences not only in the dynamics of regular versus irregular afferent activity (i.e., irregular afferents have higher sensitivities and phase leads as shown in Fig. 1b), but also in their information coding (Fig. 1c). In monkeys, regular canal afferents transmit twofold more information and are twice as sensitive for detecting head motion compared to irregular afferents [46]. Thus, regular and irregular canal afferents essentially comprise two parallel information channels, one which encodes high-frequency stimuli with higher gains (i.e., irregular afferents), the other which transmits information about the detailed time course of the stimulus over the behaviorally significant frequency range (i.e., regular afferents). Interestingly, a different coding strategy is used by otolith afferents. Irregular otolith afferents are far more sensitive than regular otolith afferents, so much so that their differences in sensitivities are effectively compensated by differences in variability [45]. As a result, neuronal thresholds are independent of both stimulus frequency and resting discharge regularity.

Our basic understanding of the existence of these two peripheral signaling streams has also contributed to the development of clinical tests. In patients, high-frequency vibrational stimuli to the skull or air-conducted sound can be used to induce ocular vestibular-evoked myogenic potentials (oVEMPs) mainly originating from the utricular macula or cervical VEMPs arising from the saccular macula [47]. These tests are applied in clinical practice to delineate otolith dysfunction in patients with posttraumatic dizziness, inferior vestibular neuritis or superior canal dehiscence syndrome [48, 49]. Experiments in rat and guinea pig have shown that this stimulation predominantly activates irregular afferents, which are also responsive to bone-conducted or airborne sound [47]. Similarly, studies in monkey, chinchilla and rat have shown that galvanic vestibular stimulation (GVS) preferentially activates irregular afferents [39, 50]. Based on the non-invasiveness of the latter method and easy application in patients, GVS has found its entry into clinical practice, and facilitates a diagnosis and dissociation between Meniere´s disease and, e.g., vestibular migraine [51].

### Vestibular neuronal typology as basis for sensory–motor processing

Central vestibular neurons play a key role in sensory–motor transformation of semicircular canal and otolith signals. Electrophysiological in vitro experiments in slices and isolated brain preparations have found two primary neuronal subtypes in the vestibular nucleus of rodents and guinea pig (type A and B neurons). These neurons distinctly differ in their resting discharge regularity, response dynamics and sets of ionic conductances [52]. While extensively characterized in guinea pig, rat and mouse, a similar dichotomy has been reported in frog (i.e., phasic and tonic neurons: [53]) and chicken (i.e., principle and elongate cells: [54]). Thus, a distinction into two vestibular subtypes appears to be the common denominator that matches the vestibular afferent organization [55].

Studies in behaving monkeys have established two main functional classes of vestibular neurons (Fig. 1a) that likely overlap with the classification scheme established in vitro [44]. The first class of neurons encodes voluntary eye movements as well as head motion. These Position-Vestibular-Pause (PVP) neurons project to extraocular motoneurons (Fig. 1a). A second class of neurons encodes head but not eye movements (i.e., vestibular-only (VO) neurons), projects to the spinal cord and is thought to mediate vestibulo-spinal reflexes for posture control (Fig. 1a). The response dynamics of the two neuronal classes in primates suggest that they receive input from two parallel sensory information streams (Fig. 1a–c). A first one, mediated by regular vestibular afferents, contains information about the stimulus’ detailed time course (stimulus estimation). The second one, mediated by irregular vestibular afferents, transmits information about the occurrence of high-frequency stimulus features (feature detection). Notably, VO neurons show dynamic properties similar to irregular afferents and respond to the high-frequency features of motion stimuli in a strongly nonlinear fashion [56–58]. This behavior is similar to that of type B neurons that have been characterized in vitro. In contrast, PVP neurons show less dynamic properties, similar to regular afferents or to type A neurons described above. However, while various properties of type A and B neurons, obtained in vitro, match very well with those of the two types of vestibular neurons recorded in vivo, to date a definitive functional description of both type A and B neurons during motion stimulation in the intact animal is lacking.

### Structure for function: Similarities versus differences in central vestibular organization

#### Semicircular canal and otolith convergence

As a general vertebrate pattern, afferent projections from vestibular endorgans largely overlap in the different central vestibular nuclei of all investigated vertebrate species including cat [59], pigeon [60], frog [61] or fish [62, 63]. This demonstrates an evolutionarily inherent absence of a sensory map. At variance with the large overlap of afferent fibers from individual endorgans, however, second-order vestibular neurons (2°VN) are organized in a hindbrain segmental (rhombomeric) pattern that is based on the motor/premotor target of a particular vestibular subgroup [5, 64]. This rhombomeric arrangement was first demonstrated in chick embryos [65, 66], and further specified in fish [67], frog [68] and mouse [69]. The discovery of this segment-specific arrangement was established by visualization of Hox gene expression patterns as well as the plain visibility of rhombomeres in various vertebrate embryos or larvae that allowed the direct mapping of distinct vestibular subgroups onto the hindbrain scaffold.

Although afferents from all vestibular endorgans overlap to a large extent in the vestibular nuclei, 2°VNs exhibit a remarkable specificity in selecting their monosynaptic afferent input; most 2°VNs receive monosynaptic inputs from only one semicircular canal (Fig. 1d–f) or one otolith organ, respectively [21]. Moreover, semicircular canal inputs combine monosynaptically with otolith afferent inputs in a spatially specific manner. For example, horizontal semicircular canal signals predominantly converge with utricular signals (Fig. 1f) and vertical semicircular canal signals with those from vertical otolith organs [70]. Thus, despite the theoretical availability of sensory signals from all vestibular endorgans, the spatial motion vector of individual afferents is largely preserved at the level of the first neuronal element in the brainstem.

In everyday life, our vestibular sensors are activated by complex multi-dimensional motion patterns that simultaneously stimulate semicircular canal and otolith organs [26, 71]. Single-unit recordings in primates have further shown that integration of semicircular canal and otolith inputs by vestibular neurons is sub-additive and characterized by frequency-dependent (nonlinear) weighting of both modalities [72]. This integration is required to discriminate tilt from translation and to ensure accurate perception and motor performance.

#### Vestibular-visual convergence

The optokinetic reflex (OKR) works together with the vestibulo-ocular reflex (VOR) to stabilize gaze. The OKR is symmetric in monkeys and humans but asymmetric in mice, gerbils and rabbits [73–75] as well as in non-mammalian species such as frogs [21]. The symmetry in primates is likely mediated by relatively stronger contributions from cortical versus subcortical pathways [76–78]. Studies in behaving mammals indicate that eye-movement-sensitive vestibular nucleus neurons command OKR eye movements and also contribute to a “velocity storage” circuitry. This network uses visual information to supplement the decaying signal from vestibular afferents during sustained head movements to encode self-motion [79, 80]. Specifically, PVP but not VO neurons are modulated during both optokinetic and vestibular stimulation in mice [81] and potentially in primates [82].

#### Vestibular-proprioception convergence

Most vestibular nuclei neurons in mice [83], rats (e.g., [84]), cats [85–87] and alert squirrel and cynomolgus monkeys [88, 89] respond robustly to passive proprioceptive stimulation as well as to vestibular inputs. Neuronal responses to combined stimulation can generally be predicted based on the linear sum of a given neurons’ individual vestibular and proprioceptive sensitivities. However, there are important differences across species. While proprioceptive responses are robust in rodents, they are less pronounced in cynomolgus monkeys [89] and completely absent in rhesus monkeys [90, 91], a difference that is likely related to species-specific adaptations in gaze strategies during exploratory behavior.

#### Multimodal convergence during active self-motion

**Fig. 2:**
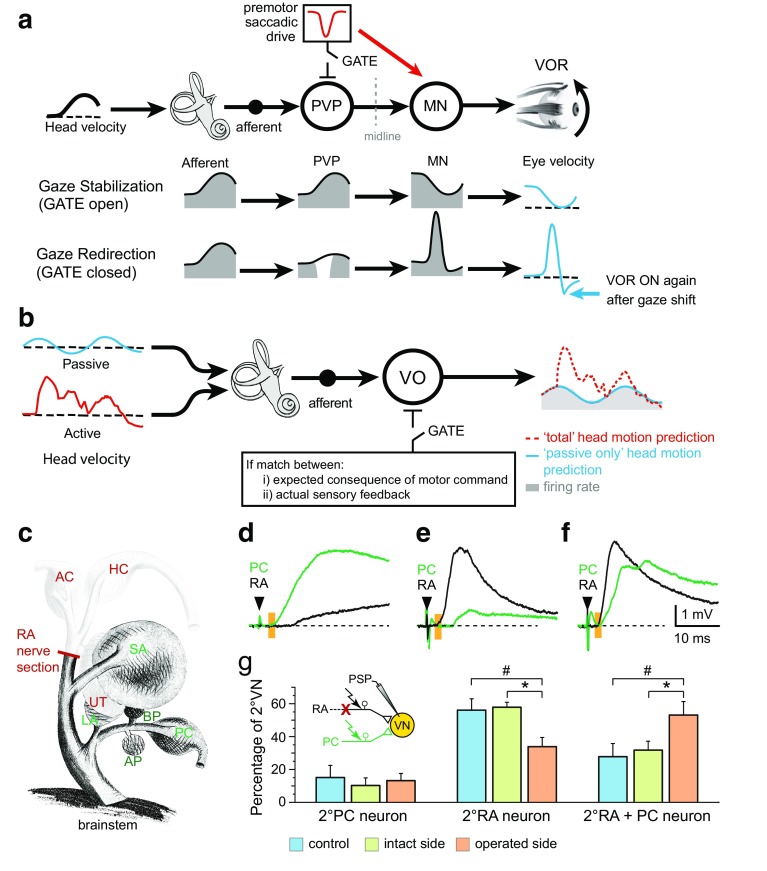
Task-dependent and lesion-induced plasticity of vestibulo-ocular reflexes during active and passive motion. **a** Schematic illustrating task-specific processing (gaze stabilization, redirection) in central vestibular PVP neurons and extraocular motoneurons. **b** Task-specific cancelation of vestibular sensory inputs by predictive signals during active (*red*) head motion in VO neurons. In contrast, vestibular sensory information is completely transmitted during passive (*blue*) head motion. **c** Schematic view of the frog VIIIth nerve with endorgans, nerve branches and site of RA nerve section. **d**–**f** Convergence of afferent inputs from the RA and PC nerve branches; monosynaptic responses were evoked in some 2°VN after stimulation of the PC nerve (*green trace*; **d**), in others after stimulation of the RA nerve (*black trace*; **e**) and in a third group after stimulation of both branches (**f**). **g** Percentages of the three types differ between controls and operated frogs and between intact and operated sides (*color-coded bars*). *Black* *arrowhead* and *orange bars* in **d**–**f** indicate stimulus and monosynaptic onset. *AC, PC, HC* anterior, posterior vertical, horizontal semicircular canal, *AP, BP* amphibian, basal papilla, *LA* lagena, *RA* ramus anterior of the VIIIth nerve, *SA* saccule, *UT* utricle, *VN* vestibular nuclei, *VO* vestibular-only neuron. **c**–**g** is based on data from [107]

Recent neurophysiological studies in primates have emphasized the importance of extra-vestibular signals in shaping sensorimotor transformations that mediate vestibulo-spinal reflexes [44, 92]. Whereas the sensitivity or firing rate of vestibular nerve afferents is virtually identical during active or passive movements (Fig. 2a), the discharge of VO neurons shows striking differences in the two conditions. Notably, while VO neurons robustly respond during passive head movements, their vestibular-related modulation is markedly attenuated during active head movements (Fig. 2b). This attenuation is behaviorally advantageous during voluntary movements, since intact vestibulo-spinal reflexes would likely be counterproductive, eliciting postural responses that would oppose intended voluntary movements.

### Neural circuits for the control of gaze: different constraints versus common principles

The circuitry underlying the VOR is the best characterized vestibular-driven pathway. In his classical studies, Lorente de Nó [93] demonstrated the existence of a three-neuronal arc that represents the most direct pathway between vestibular afferents and eye muscles. Thereafter, studies using many different vertebrate species to probe different aspects of this reflex circuitry, have established that the basic functional organization of the VOR has remained virtually unchanged since it evolved in early vertebrates [64]. In fact, this reflex pathway is remarkably well conserved in vertebrates spanning from fish to mammals with regard to segmentally arranged neuronal phenotypes, employed neurotransmitters, differential organization of the horizontal and vertical angular VOR and the conjugation of eye movements [5].

The relative simplicity of the VOR makes it an excellent model system for studies that try to bridge the gap between neuronal circuits and behavior. It is arguably our fastest behavior [94], consistent with the synaptic and axonal delays of the three-neuronal arc. The VOR shows a remarkable compensatory gain (eye velocity/head velocity = 1) as well as minimal phase lag over the physiological relevant range of head movements [94, 95]. The results of single-unit recordings in monkeys have provided insight into how the VOR effectively stabilizes gaze across a wide range of head velocities and frequencies experienced in everyday life [44]. Experiments in cats, monkeys and humans have shown that the efficacy of the VOR depends on the actual behavioral goal. While the VOR is compensatory when the goal is to stabilize gaze, it is attenuated or even suppressed when gaze (eye/head and or body movements) is redirected toward a target of interest.

The discovery of VOR efficacy as a function of the behavioral goal has particularly benefitted from single-unit analyses of multimodal integration in vestibular neurons of monkeys [44]. While vestibular afferents robustly encode head motion regardless of behavioral goals, responses of vestibular nucleus neurons (i.e., PVPs) are attenuated during gaze redirection (Fig. 2a). The neurophysiological bases of this suppressive influence are the well-characterized inhibitory projections from the brainstem premotor saccadic and pursuit pathways to the vestibular nuclei. Because an efference copy of the motor command to voluntarily redirect gaze suppresses the responses of PVP neurons, the efficacy of the VOR pathway critically depends on the actual gaze strategy during a particular situation (Fig. 2a). A more general role of motor efference copies in gaze stabilization is suggested by the findings in amphibians where copies of spinal locomotor commands directly drive compensatory eye movements during active motion [96, 97]. Given the neuronal connectivity between the spinal rhythm generator and the extraocular motor nuclei, it is very plausible that such connectivity represents an evolutionarily ancient pathway that might still have functional relevance in mammalian species. However, independent of its relevance in other vertebrates, it offers insight into a basic conceptual design that is important for understanding the role of the vestibular system in general.

In clinical practice, VOR testing has become highly important to detect deficits of the semicircular canals. The clinical head impulse test (HIT) for the horizontal semicircular canal was described for the first time by Halmagyi and Curthoys [98]. Today, video-based horizontal and vertical HIT recordings are routinely applied to measure VOR gain of all three semicircular canals to potentially detect compensatory saccades (so-called covert/overt saccades) in patients with vestibular disorders [99, 100]. VOR testing thereby complements caloric irrigation, because both methods test different frequency spectra of vestibular afferents (high versus low frequency). In the clinical context, VOR testing is most important to differentiate a peripheral versus central origin of an acute vestibular syndrome [101, 102].

### Principles of vestibular compensation and motor learning

Scientists and clinicians have long been fascinated by the capability of animals and humans to recover from the behavioral deficits after a loss of labyrinthine function [3, 10]. In the absence of a regrowth of the sensory organ, any functional improvement must be due to a reorganization of the signal processing in the central nervous system. Our current knowledge of the physiology of the compensation process has greatly benefitted from the employment of different animal models [103–105]. For example, work in rodents and guinea pigs has revealed a reduction of GABAergic commissural inhibition on the impaired side, changes in the strength of cerebellar inputs to the vestibular nuclei, as well as a shift toward more linear response properties of the deafferented type B vestibular neurons and an inverse pattern on the intact side [55]. Experiments in monkeys suggest a small but significant relative increase in the proportion of irregular afferents of the vestibular nerve on the contralesional side that parallels the shift in response properties observed in rodents and guinea pigs [106]. Experiments in frogs have further shown that a partial lesion of the vestibular nerve (Fig. 2c) causes synaptic reorganization of remaining ipsilateral afferent inputs onto deafferented 2°VNs (Fig. 2d–g) and a considerable reduction of the commissural inhibition at the expense of a modified vestibular reflex directional specificity [104, 107]. Across a variety of mammalian species including humans, monkeys, cats, guinea pigs and mice, VOR compensation is nearly complete for rotations toward the contralesional side, but incomplete for rotations toward the ipsilesional side, particularly for more dynamically challenging stimuli [105, 108]. Consistent with behavioral responses, experiments in behaving monkeys have shown that the responses of PVP neurons decrease immediately following a unilateral vestibular loss, but subsequently recover within a few weeks to reach values close to those measured before the lesion [109].

Findings across a variety of animal models have further established that a common denominator of vestibular compensation is the induction of homeostatic plasticity. For example, postural recovery in frogs after unilateral labyrinthectomy depends on an altered efficacy of spinal reflexes provided that body-weight-supporting limb proprioceptive inputs are available [55]. Interestingly, while this is the case in terrestrial vertebrates, aquatic species lack these signals and instead develop scoliotic deformations likely due to a permanently manifested vestibular asymmetry [110]. Similarly, compensation in monkeys is mediated by rapid dynamic reweighting of inputs from different modalities (i.e., extra-vestibular proprioception and motor efference copy signals *versus* vestibular signals) at the level of vestibular nucleus neurons [109, 111–113]. Thus, multimodal integration is dynamically regulated in the vestibular system, in a manner that suggests a causal role for homeostatic plasticity in VOR compensation. This strategy appears common across vertebrates, providing a neural substrate for rehabilitation approaches currently used by clinicians to treat patients. Potential strategies for physical therapy after unilateral vestibular damage thus may include activities such as the Cawthorne-Cooksey exercises that involve a progression of increasingly complex head and body movements (reviewed in [114] ), active VOR gain adaptation [115], neck muscle vibration [116] or an increased use of visual reference frames [117].

Plasticity within vestibular pathways also plays an essential role in fine-tuning the coordination and accuracy of the VOR in response to environmental or developmental alterations. For example, adaptive changes in VOR performance are required to compensate for the mismatch between visual and vestibular stimuli caused by the magnification of corrective lenses worn during common visual conditions [118, 119]. Such a gain control of the VOR is implemented by the prominent feed-forward cerebellar circuitry [21]. This circuit is highly plastic and provides the basis for the cerebellar contribution to motor learning. Experiments in cats and monkeys have further established that plasticity within the floccular complex of the cerebellum initially drives VOR adaptation, which in turn triggers longer term synaptic changes in floccular target neurons within the vestibular nuclei [120, 121]. In addition, in vivo and in vitro studies suggest that synaptic plasticity occurs within non-cerebellar VOR pathways alongside synaptic changes within the cerebellum [122–125]. Thus, it is likely that plasticity processes for gain modifications of vestibular reflexes are distributed features at multiple sites that allow guiding adaptations to maintain VOR accuracy.

### Higher level processing and perception

Vestibular information is not only required for reflexive motor reactions but also vital for cognitive functions such as perception of self-motion, spatial orientation and body representation [126]. Single-unit studies in non-human primates have provided insight into the computations performed by the cerebellum and cortex. In addition, neuroimaging using caloric and galvanic vestibular stimulation have provided insight into how these higher order areas process vestibular stimuli [127].

The vestibular cerebellum integrates vestibular and extra-vestibular information to make fundamental contributions to self-motion perception. The nodulus–uvula (lobules X and IX) is thought to create an internal model of spatial orientation that accounts for the physics of our world. Consistent with theoretical predictions [128], some neurons combine otolith and semicircular canal inputs to distinguish tilt from translation [129]. In addition, the vestibular cerebellum integrates vestibular and proprioceptive inputs to represent head and body-in-space motion in two separate streams [130]. Specifically, neurons in the deep cerebellar nuclei (i.e., fastigial), which receive inputs from the anterior cerebellar vermis, encode body motion independently of head motion. Moreover, these neurons selectively and dynamically encode passive head and body motion relative to space, suggesting that the cerebellum computes an internal model of the expected sensory consequences of active head motion to selectively cancel respective responses [131]. This mechanism is likely responsible for the attenuation of active motion observed in early vestibular processing (i.e., see Fig. 2b).

Ascending projections from the vestibular nuclei and vestibular cerebellum terminate in regions of the thalamus (reviewed in [132, 133] ), which in turn project to the cortex. In contrast to most other sensory systems, there is not a single primary cortical area devoted to vestibular signals. Instead, vestibular-related activity is found in multiple regions, including the parieto-temporal, frontal, somatosensory and extrastriate visual cortices (reviewed in [134] ). Notably, most neurons in these areas receive converging visual and/or somatosensory inputs. Among these areas, the parietoinsular vestibular cortex (PIVC) is generally considered as primary vestibular cortex since (i) PIVC neurons respond to vestibular input [135–137], (ii) stimulation of this area produces vestibular sensations in humans [138], (iii) lesion of PIVC impairs perception of the subjective visual vertical [139] and (iv) cerebral blood flow of the PIVC area increases during vestibular stimulation [140–142]. Numerous studies have also focused on how vestibular processing in the dorsal medial superior temporal cortex (MSTd) contributes to our perception of self-motion (reviewed in [143]). The transmission of self-motion information from these cortical areas to entorhinal and perirhinal cortices and the hippocampus is thought to play a critical role in spatial cognition and navigation (reviewed in [144]). In fact, patients with a bilateral vestibulopathy show deficits in spatial orientation along with a markedly reduced hippocampal volume [145]. The contribution of the vestibular system in the pathophysiology of disorders of spatial attention such as neglect is increasingly recognized and can be used for rehabilitation [146, 147].

## Advantages of different animal models

As illustrated in the previous chapters, studies using a wide variety of vertebrate species have been essential for furthering our knowledge of how the sensory organs in the inner ear detect head motion in space, how receptor cells transduce motion into voltage signals, and how the brain encodes and integrates these motion-related signals for accurate behavior and perception during self-motion. Notably, while some species are better suited than others to answer scientific questions on the organization and role of the vestibular system, others are better suited to establish the neural circuitry mediating higher order functions such as vestibular cognition. Vestibular research has particularly benefitted from its positioning at the intersection between basic and clinical science. For example, neural pathways mediating vestibular-driven reflex behaviors such as the VOR are relatively simple, and collective knowledge from studies using a wide variety of species have provided an excellent framework for understanding the physiology underlying clinical syndromes.

Overall, we argue that in the future a reasonably balanced spectrum of animal models will continue to be required to increase our understanding of the vestibular system. Studies of ontogenetic aspects and developmental assembly of appropriate neural connections will require further work in model species that allow easy access to embryonic stages. For instance, genetic approaches and developmental manipulations can be combined with physiology in vertebrates such as fish, frog, chicken or mouse, even though recordings are rather difficult to perform in embryonic and early post-embryonic birds or mammals [29]. With respect to cellular details of vestibulo-motor signal processing, electrophysiological recordings in slice preparations of rodents and guinea pigs have revealed important fundamental principles [55] and new innovative approaches including multichannel electrophysiological recordings and optical imaging will provide greater accessibility to population coding in vestibular structures. In addition, complementary studies in more “exotic” species will continue to contribute to reveal general concepts of sensory–motor transformation in vertebrates such as the Axolotl with its legendary regenerative capacity [148], turtles that allow robust in vitro experiments in an amniote vertebrate species [149], or flatfish that exhibit a substantial VOR reorganization during the transition from bilateral-symmetric free-swimming larvae to asymmetric bottom-dwelling adults [150].

For studies of evolution and adaptation in the vestibular system and its cellular components, as well as the respective computations performed by early vertebrate ancestors or higher order cortical processing, an even wider range of vertebrate species is required [4, 67]. This is because central processing of vestibular signals depends on both intrinsic membrane and emerging network properties. Accordingly, deciphering their interactions requires experimental models with intact nervous systems that also provide the researcher with an experimental accessibility necessary to manipulate the respective neural circuitry. In the past, the isolated guinea pig or frog whole brain [55] substantially contributed to our understanding of underlying computations, with further improved probing of morpho-physiological aspects in recently developed semi-intact amphibian preparations [151]. We speculate that future mouse or zebrafish lines with genetically expressed calcium ion sensors will yield further enhanced accessibility to vestibulo-motor networks for in vivo recordings of cell and circuit activity.

In contrast, work in more advanced mammalian species including non-human primates will be required to further our understanding of how these circuits give rise to perception, cognition and behavior under normal conditions—knowledge essential for developing more effective health protocols to diagnose and treat the debilitating symptoms of vestibular disorders in patients. Decades of electrophysiological observations in non-human primates have already provided key insights as to how vestibular-driven sets of motor behavior and perception arise from neural circuit activity (reviewed in [44, 92]). Notably, primates will be the model of choice for studying the mechanisms that provide perceptual stability and accurate motor performance during common yet complex behaviors, such as combinations of voluntary head motion and locomotion. Further, while some basic brain circuits are preserved, many of the neural circuits related to higher cognitive functions differ between non-mammalian species and humans. By comparison, since the brain organization of humans and non-human primates are remarkably similar, especially with regard to the cerebellum and cerebral cortex, this animal model is particularly well suited for studies aimed at understanding the higher level organization of vestibular processing and motion perception [44, 92, 143, 152].

Finally, it is important to emphasize that studies across a wider range of species will be required to facilitate translational vestibular research progress. For studies focused on efficient, high-throughput drug discovery [153], understanding general circuit organization or multimodal interactions and the impact of motor efference copies for sensory–motor transformations [110], non-mammalian species are highly relevant and convenient model systems. In addition, drug treatments initially developed in species such as mice can fail when translated to humans, emphasizing the value of non-human primates in translational research. In summary, a comparative approach based on studies across a variety of vertebrate species, each with particular advantages for defined scientific questions, remains necessary to maximize our understanding of the vestibular system and its pathophysiology.
